# Longitudinal T2 Mapping and Texture Feature Analysis in the Detection and Monitoring of Experimental Post-Traumatic Cartilage Degeneration

**DOI:** 10.3390/life11030201

**Published:** 2021-03-05

**Authors:** Marc Sebastian Huppertz, Justus Schock, Karl Ludger Radke, Daniel Benjamin Abrar, Manuel Post, Christiane Kuhl, Daniel Truhn, Sven Nebelung

**Affiliations:** 1 Department of Diagnostic and Interventional Radiology, Aachen University Hospital, 52074 Aachen, Germany; mhuppertz@ukaachen.de (M.S.H.); mpost@ukaachen.de (M.P.); ckuhl@ukaachen.de (C.K.); dtruhn@ukaachen.de (D.T.); 2 Department of Diagnostic and Interventional Radiology, Medical Faculty, University Dusseldorf, 40225 Dusseldorf, Germany; justus.schock@med.uni-duesseldorf.de (J.S.); ludger.radke@med.uni-duesseldorf.de (K.L.R.); DanielBenjamin.Abrar@med.uni-duesseldorf.de (D.B.A.)

**Keywords:** cartilage, focal cartilage defect, posttraumatic osteoarthritis, T2 mapping, texture analysis

## Abstract

Background: Traumatic cartilage injuries predispose articulating joints to focal cartilage defects and, eventually, posttraumatic osteoarthritis. Current clinical-standard imaging modalities such as morphologic MRI fail to reliably detect cartilage trauma and to monitor associated posttraumatic degenerative changes with oftentimes severe prognostic implications. Quantitative MRI techniques such as T2 mapping are promising in detecting and monitoring such changes yet lack sufficient validation in controlled basic research contexts. Material and Methods: 35 macroscopically intact cartilage samples obtained from total joint replacements were exposed to standardized injurious impaction with low (0.49 J, n = 14) or high (0.98 J, n = 14) energy levels and imaged before and immediately, 24 h, and 72 h after impaction by T2 mapping. Contrast, homogeneity, energy, and variance were quantified as features of texture on each T2 map. Unimpacted controls (n = 7) and histologic assessment served as reference. Results: As a function of impaction energy and time, absolute T2 values, contrast, and variance were significantly increased, while homogeneity and energy were significantly decreased. Conclusion: T2 mapping and texture feature analysis are sensitive diagnostic means to detect and monitor traumatic impaction injuries of cartilage and associated posttraumatic degenerative changes and may be used to assess cartilage after trauma to identify “cartilage at risk”.

## 1. Introduction

Associated with knee symptoms and dysfunction, focal cartilage lesions are common in the general population. Hjelle et al. reported (osteo)chondral lesions (of any type) in 61% of their patients undergoing knee arthroscopy [1]. These findings were confirmed by other studies, too [2,3,4]. The tissue’s limited intrinsic healing capacity and the progressive nature of cartilage lesions warrant additional diagnostic and therapeutic efforts to prevent osteoarthritis (OA) and its great socioeconomic and personal disease burden.

While the aetiology of focal cartilage lesions is multifactorial, focal cartilage lesions are often the result of trauma [5,6]: Meniscus and anterior cruciate ligament (ACL) injuries bring about instability and predispose the joint to cartilage lesions [5]. Similar dispositions are incurred by patellar dislocations. The prevalence rates of cartilage lesions in the patellofemoral joint are 71%, 82%, and 97% in acute, recurrent, and chronic dislocators, respectively [6]. Other aetiologic factors are fractures, soft-tissue injuries, and repetitive microtraumatizations that result in surface incongruity, altered joint kinematics, and chronic degenerative changes, thereby predisposing to cartilage lesions, too. Consequently, posttraumatic OA (PTOA) accounts for nearly 12% of all cases of symptomatic OA in the United States [7].

Due to its high soft tissue contrast and spatial resolution, non-invasiveness, and lack of radiation, Magnetic Resonance Imaging (MRI) is clearly the most powerful diagnostic tool of contemporary clinical medicine and the superordinate standard imaging modality for suspected joint and cartilage disorders [8,9,10]. However, numerous studies have indicated the limitations of clinical-standard morphologic MRI techniques in the detection of cartilage lesions with variable sensitivities of 45% to 74% [11,12]. With the positive predictive value equally variable, morphologic MRI techniques are (i) not able to reliably indicate the presence (or absence) of cartilage lesions and (ii) particularly limited in detecting early, potentially reversible cartilage lesions. Consequently, quantitative MRI techniques such as T2 and T1ρ mapping have received ever-increasing scientific and clinical attention over the last decades [13,14]. These techniques quantify biophysical tissue properties on the compositional and ultrastructural level beyond mere morphology. Widely available on clinical MRI scanners and conveniently acquired with an additional scan time of 5 min, the addition of a T2 mapping sequence to a routine imaging protocol improved sensitivity in the detection of (early) cartilage lesions [15]. T2 mapping is a robust, clinically and scientifically well-validated, and commonly used technique to assess cartilage status [13,14]. Moreover, T2 mapping is closely associated with relevant structural and compositional tissue features such as collagen content, collagen network organization and integrity, and water content [16]. Consequently, a solid body of evidence has been collected that indicates the potential of T2 mapping in evaluating posttraumatic cartilage changes [17,18,19,20,21,22,23].

Instead of merely quantifying T2 values of the superficial and deep cartilage zones in a pixel-wise manner, recent approaches have relied on more comprehensive post hoc approaches for image analysis such as texture feature analyses. During cartilage degeneration, collagen network integrity and proteoglycan content are lost [24]. The increasing degrees of tissue disruption and disorganization translate to altered spatial distributions of T2 and may be quantified as markers of heterogeneity based on textural features. In degenerated cartilage, T2 values tend to be elevated with greater local heterogeneity [17,25] as has been demonstrated for cartilage lesions [26], symptomatic OA and patients at risk of developing OA [25,27,28], and after ACL injury [29].

Despite this wealth of clinical knowledge, a basic understanding of the posttraumatic degenerative changes in cartilage and their imaging correlates is lacking. The present study’s objective was to contribute to this understanding by bringing together intact human articular cartilage, standardized injurious impaction loading with variable impaction energies, and T2 mapping and post hoc texture feature analysis. To this end, (histologically referenced) intact cartilage tissue was subject to impaction loading using a drop-tower device as an established model for inducing posttraumatic degenerative changes [30,31,32], and imaged longitudinally to study these changes as a function of time and impaction energy, i.e., trauma severity. Our hypotheses were that (i) variable impaction energies induce variable progressive posttraumatic degenerative changes in cartilage and that (ii) these changes are reflected by the T2 maps and associated descriptive statistics and texture features.

## 2. Materials and Methods

### 2.1. Cartilage Sample Preparations

**Figure 1:**
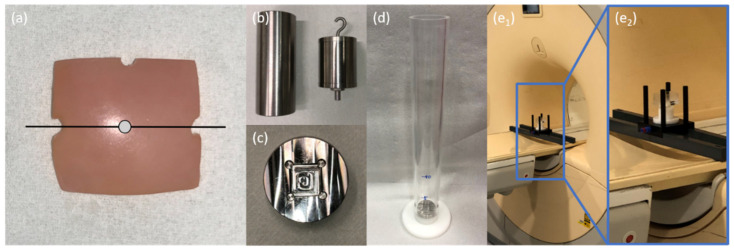
Details of cartilage sample preparation and subsequent injurious impaction loading. (**a**) Top view of a representative osteochondral sample. Notches at the 3 and 9 o’clock positions indicate the mid-sagittal plane (black line), while the sample centre point is defined as the intersection of this mid-sagittal plane and its perpendicular along the notch at the 12 o’clock position (grey dot). (**b**) Weights of 1000 g (left) and 500 g (right) equipped with hook and 5 mm-tip to induce standardized injurious impaction. (**c**) Metallic sample plate with flat recess to fix cartilage samples during impaction by preventing lateral displacement. Seeming distortions are secondary to the milling process and room lights. (**d**) Cylindrical drop-tower device with metallic sample plate and indications of height. Impaction energy levels were regulated by adjustment of weight and height. Details of the assembled measurement framework at variable magnifications (blue boxes) within the scanner’s bore. The framework included support beams mounted on the MRI table (**e_1_**) and a transparent sample box (containing the cartilage samples, (**e_2_**)) for reproducible positioning of sample and coil (not shown).

Following informed consent and Institutional Ethical Review Board approval (Ethical Committee, RWTH Aachen University, Germany, AZ EK 157/13), we obtained human articular cartilage-bone samples from 24 patients undergoing total knee replacement surgery at our institution (10 male, 14 female; mean age 63.4 years [range: 53–89 years]) [33,34,35,36,37,38,39]. Primary OA of the knee as determined radiographically and clinically was defined as the inclusion criterion, while all forms of secondary OA as well as previous trauma and/or surgery, and other bone and joint diseases were defined as exclusion criteria. Immediately after intraoperative excision, cartilage-bone material was collected in sterile Dulbecco’s modified Eagle’s medium (DMEM) containing 100 U/mL penicillin, 100 µg/mL gentamycin, and 1.25 U/mL amphotericin B (all from Gibco-BRL, Gaithersburg, MD, USA). Subsequent preparations were carried out as before [32,37,38]. To maintain topoanatomic consistency, only cartilage-bone material from the lateral femoral condyle was included. Samples were cut to standard size (length × width: 15 × 15 [mm]) and any cancellous bone was removed while preserving the subchondral lamella and keeping the surface as plain as possible. Samples were then graded according to the Outerbridge classification that assesses the tissue’s macroscopic appearance [40]. Only Outerbridge-grade 0 samples, i.e., normal cartilage without softening or swelling, were included. For reference purposes, three notches were created using a rongeur, i.e., two notches at opposing sample sides to define the mid-sagittal imaging plane and a third notch to define an orthogonal plane. The intersection of these planes was defined as the sample centre point (Figure 1a). Additionally, macroscopically similar cartilage tissue immediately adjacent to the actual cartilage sample was prepared along the mid-sagittal plane to assess baseline histologic characteristics of the cartilage-bone material.

Before the study, minimum sample size had been projected using a dedicated online tool (https://www.statstodo.com, accessed 3 February 2018). Based on comparable studies [25,27] and assuming a statistical power of 0.9, a type-I error probability of 0.05, a maximum inter-group difference of 0.6, and an intra-group standard deviation of 0.5, we determined a minimum sample size of 28 (Cohen’s effect size model f2). Finally, 35 samples were thus prepared and transferred to 12-well plates filled with DMEM and additives as above.

### 2.2. Cartilage Sample Impaction

Following their preparation, cartilage samples were allocated to three groups, i.e., unimpacted controls (CONT, n = 7), low impact (LIMP, 0.49 J, n = 14), and high impact (HIMP, 0.98 J, n = 14). A custom-made drop tower device was used to standardize impaction energy levels as before [32] (Figure 1b–d). Briefly, its specifications (height 33 cm; diameter 4 cm) allowed dropping standard cylindric iron weights (500 g or 1000 g, equipped with a 5-mm-diameter tip) from defined heights. In this study, these weights were dropped from a height of 100 mm, thereby exposing the cartilage samples to two impaction energy levels of 0.49 J and 0.98 J (Table 1) [32,41]. Velocity of impact (v) was determined by drop height (h) (Equation (1)) and energy I by mass (m) and height (h) (Equation (2)), where *g* is gravity-induced acceleration.

V = (2*g*h)^1/2^ (1)

E = m*g*h (2)

These two different impaction energy levels created “mild” (LIMP) and “severe” (HIMP) structural and compositional damage in cartilage [32,41]. To ensure localized impaction at the sample centre point without lateral displacement, a custom-made metallic sample plate with a dedicated recess (depth: 2 mm, width × height: 16 mm) confined and fixed the cartilage samples on all sides (Figure 1c). After impaction, the weight’s tip was left to rest on the samples for 5 s to ensure constant compression conditions [42].

### 2.3. MRI Measurements

**Figure 2:**
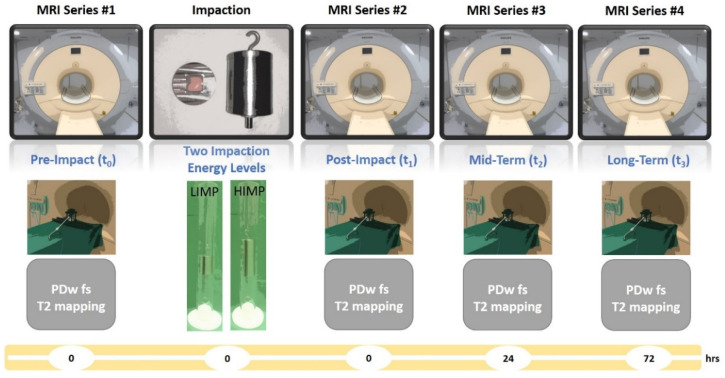
MRI series timeline. Cartilage samples were imaged immediately before (t_0_) and after impaction (t_1_) as well as after another 24 h (t_2_) and 72 h (t_3_). The imaging protocol was completed for each MRI series (grey boxes). Unit of timeline (yellow) is hours.

Pre- and post-impaction MRI measurements were performed on a clinical 3.0 T MRI scanner (Achieva, Philips, Best, The Netherlands) within 6 h after sample preparation. Serial MRI measurements were performed just before (t_0_) and immediately after impaction (t_1_) as well as 24 h (t_2_) and 72 h (t_3_) after impaction (Figure 2).

For reproducible positioning of cartilage samples in the MRI scanner, a validated MRI-compatible device [39,43] was used. The device’s lower frame contained the transparent sample box with the cartilage samples and was mounted on the patient table by means of dedicated support beams (Figure 1e). Thus, imaging was performed close to the scanner’s iso-centre with the sample surfaces and mid-sagittal planes parallel to the main magnetic field B_0_. A modified single-channel receive-only prostate coil (BPX-40 disposable endorectal coil, Medrad/Bayer, Germany) without the inflatable balloon tip was used for imaging and circumferentially enclosed the sample box. Radiofrequency pulses were applied via the scanner’s in-built body coil. Two cartilage samples were imaged at a time using morphologic and quantitative MRI (Table 2). Briefly, following scout views, Proton Density-weighted sequences were acquired in the sagittal, coronal, and axial orientations. These sequences were used to guide the T2 mapping sequence along the mid-sagittal plane as indicated by the notches at opposite sample sides. Notably, off-the-shelve fish oil capsules (1000 mg) were attached to the sample box for identification purposes. Total imaging time per MRI series was 11 min 40 sec and total imaging time per sample pair was 46 min 40 s. Measurements were carried out at room temperature, which was monitored during one representative MRI series (20.3 ± 0.5 °C). After the respective MRI series, the cartilage samples were retrieved, placed in DMEM and additives, and cultured under standard conditions (5% CO_2_; 37 °C; humid air) in a standard incubation unit. Culture medium was changed after each MRI series.

### 2.4. MRI Data Analysis

The T2 maps were generated in a pixel-wise manner using customized mono-exponential fitting routines implemented in MATLAB (MatlabR2020a, Natick, MA, USA) as before [35,39]. For fitting, only echo times 2–7 (i.e., echo times < 60 ms with the exception of the first echo to avoid artefacts secondary to stimulated echoes) were included because of the insufficiently low signal-to-noise ratios beyond 60 ms. Cartilage samples were segmented manually by delineating the sample outlines on a moderately T2-weighted morphologic image (TE = 41.9 ms). To prevent partial volume effects and subsequent T2 quantification errors, segmentation outlines were delineated conservatively, i.e., only pixels that certainly constituted cartilage tissue were included. Besides the entire sample’s cross-section, additional regions-of-interest (ROIs) were defined by automatically dividing the samples into the superficial and deep cartilage layers. Based on another customized MATLAB routine and the segmentation outlines, sample height was determined along the sample’s entire width to automatically create two equally thick layers. M.S.H. (2 years of experience in musculoskeletal imaging) performed the manual segmentations that were quality checked by S.N. (9 years of experience in musculoskeletal imaging). For each ROI, summary statistics, i.e., means ± standard deviations, and texture variables, i.e., variance, contrast, homogeneity, and energy, were calculated using customized MATLAB routines based on earlier approaches [44,45]. Information on the spatial arrangement of T2 values were derived using gray-level co-occurrence matrices (GLCMs) for each texture feature. GLCMs serve to tabulate the frequency with which pixel value combinations are present and may be used to determine numerous texture features. Separate GLCMs were determined for each texture variable with an offset of a single pixel based on vertical, horizontal, and angular orientations, i.e., 0°, 45°, 90°, and 135° [45]. Of note, orientation-dependent inputs were averaged [25]. **Metrics of contrast**, i.e., contrast and homogeneity, **metrics of orderliness**, i.e., energy, and **statistical metrics**, i.e., variance, were calculated [17]:

1. **Contrast** assesses the extent of local variation. Cartilage areas with high contrast values display strong contrasts, i.e., pronounced differences between the highest and lowest T2 values.
2. **Homogeneity** serves as a measure of uniformity by indicating similarities between pixels and their neighbours. Cartilage areas with mostly similar T2 values have high homogeneity values.
3. **Energy** as computed based on the GLCM provides a measure of uniformity and orderliness. Cartilage areas with high energy values display similar T2 values and small T2 value differences in neighbouring pixels.
4. **Variance** is a measure of local variation around the mean. High variance values indicate high heterogeneity and large differences in T2 values, i.e., variation from their mean.

### 2.5. Histologic Reference Analysis

Following the last MRI series at t_3_, the cartilage samples and the adjacent cartilage tissue underwent standard histological work-up [32,46]. Samples and adjacent tissue were simultaneously decalcified and fixed in Ossa fixona (Diagonal, Muenster, Germany), sectioned along the mid-sagittal plane (or parallel to it), embedded in paraffin, cut to 5-µm sections, and stained with haematoxylin/eosin and Safranin O. Histologic evaluation, including documentation, was performed on a standard light microscope (Leica DM/LM-P, Wetzlar, Germany).

Baseline cartilage status of the **adjacent cartilage tissue** was assessed semi-quantitatively according to the Mankin classification of cartilage degeneration [47]. Two investigators, i.e., M.S.H. and S.N. with 3 and 11 years of experience in musculoskeletal histopathology, individually assessed each tissue specimen. The Mankin classification assesses tissue structure (score, 0–6), cellularity (score, 0–3), proteoglycan staining intensity (score, 0–4), and tidemark integrity (score, 0–1). Based on each tissue feature’s individual score, the Mankin sum score (MSS) gives the cumulative score of degeneration. Ranging from 0–14, lower or higher MSSs indicate less or more severe signs of histologic degeneration, respectively. In case of differing scores, respective histologic sections were discussed until consensus was reached. Of note, only grossly intact cartilage samples with MSS of 0–4 were included.

Following exposure to impaction or control conditions, **cartilage samples** were assessed semi-quantitatively based on the Mankin classification. Posttraumatic cartilage changes such as alterations in tissue structure, i.e., clefts and other signs of surface disintegration, as well as composition, i.e., Safranin O de-staining, were noted.

### 2.6. Statistical Analysis

Statistical analysis was performed by MSH and SN using GraphPad Prism Software (Version 8.0.2, San Diego, CA, USA). Not assuming normal distributions, absolute T2 values and texture features were compared as a function of time (i.e., between t_0_, t_1_, t_2_, and t_3_) using Friedman’s test followed by Dunn’s post hoc test. Accordingly, relative changes for each measure (Δ_x_) at t_x_ were determined in reference to t_0_ and calculated using (Equation (3)):

Δ_x_ = ((T2t_x_/T2t_0_) − 1) × 100 [%] (3)

The different impaction energy levels and control conditions at t_x_ were comparatively evaluated using the Kruskal–Wallis test. Group-wise, one-way ANOVA followed by Tukey’s post hoc tests (wherever appropriate) were used to compare relative changes Δ_x_ as well as histological changes of cartilage structure and proteoglycan content. Results are displayed as mean ± standard deviation. Due to this study’s exploratory character and the large number of comparisons involved, the level of significance was set to *p* ≤ 0.01 to reduce the number of statistically significant, yet clinically and scientifically (most likely) insignificant findings.

## 3. Results

All 35 samples underwent full MRI and histologic reference evaluation.

### 3.1. Macroscopic Reference Evaluation

**Figure 3:**
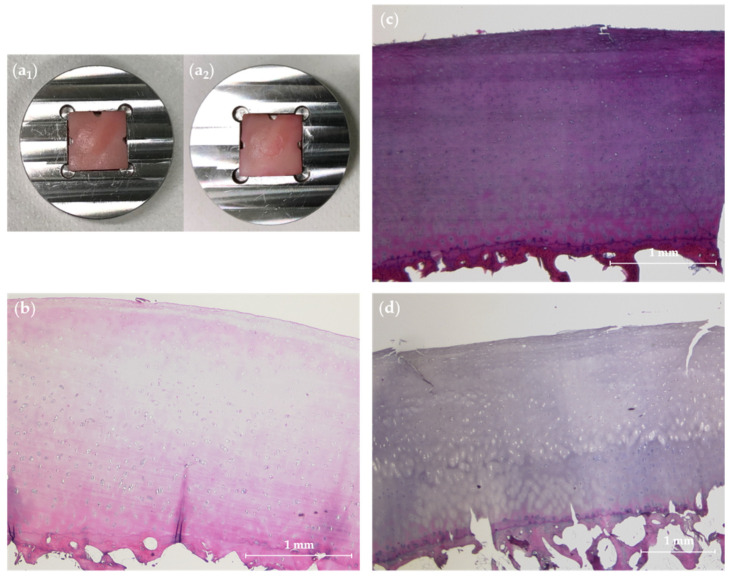
Representative macroscopic and histologic findings after injurious impaction of cartilage samples. (**a**) Representative cartilage sample positioned in metallic sample plate before (**a_1_**) and after (**a_2_**) impaction at high energy (HIMP). Visual inspection revealed clearly visible dent at the area of impaction. (**b**) Histologically grossly intact adjacent cartilage tissue with slight hypercellularity (Mankin sum score 2), but otherwise normal, indicates gross cartilage integrity at baseline. (**c**) Cartilage sample after exposure to low-energy impaction (LIMP) with a single cleft at the tissue surface, yet without any other histologic signs of posttraumatic cartilage degeneration. (**d**) Cartilage sample after HIMP exposure with distinct surface clefts marking the area of impaction and adjacent surface irregularities. Haematoxylin/eosin staining. Scale bars indicate 1 mm.

Macroscopic evaluation revealed no fracturing of the subchondral bone lamella in any sample. However, considerable dents corresponding to the metallic tip’s geometry were observed at the impaction site following HIMP exposure (11/14 samples) (Figure 3a), while no such marks were observed after LIMP exposure or in controls.

### 3.2. Histologic Reference Evaluation

Histologic assessment revealed that **adjacent cartilage tissue** was grossly intact as indicated by mean MSSs of 1.8 ± 0.8 (range, 0–3). Baseline cartilage status was dominated by slight signs of histologic degeneration such as surface fibrillation, focal hypercellularity or slight Safranin-O de-staining, translating to MSSs of 1 or 2 in most tissue specimens.

**Cartilage samples** harvested after impaction and/or standard incubation revealed impaction energy level-associated tissue damage. Even though not significant, surface disintegration was higher with higher impaction energy levels (Table 3). Similarly, non-significant differences were observed for Safranin O de-staining that tended to be more severe after HIMP than LIMP exposure. Qualitatively, Figure 3b–d gives representative histologic findings.

### 3.3. MRI Data—Descriptive Statistics

For T2, global and zonal changes were found as a function of time and impaction energy level (Table 4).

In controls, T2 values remained largely constant even though, by trend, a slight and non-significant progressive increase was noted for all ROIs, from 33.3 ± 5.1 ms (t_0_) to 35.1 ± 7.4 ms (t_3_) (*p* = 0.093, entire cartilage sample), from 37.0 ± 6.4 ms (t_0_) to 39.0 ± 8.8 ms (t_3_) (*p* = 0.180, superficial layer), and from 29.4 ± 5.1 ms (t_0_) to 31.1 ± 6.6 ms (t_3_) (*p* = 0.180, deep layer). After LIMP exposure, T2 values were significantly increased in all ROIs, from 32.0 ± 2.4 ms (t_0_) to 40.3 ± 5.2 ms (t_3_) (*p* < 0.001, entire cartilage sample), from 38.2 ± 4.3 ms (t_0_) to 47.5 ± 6.1 ms (t_3_) (*p* < 0.001, superficial layer), and from 26.1 ± 2.8 ms (t_0_) to 33.9 ± 6.9 ms (t_3_) (*p* < 0.001, deep layer). Post hoc analysis revealed these differences to be significant between t_0_ and t_2_ and between t_0_ and t_3_ (Supplementary Table S1). Similarly, after HIMP exposure, T2 values were significantly increased in all ROIs, too, from 35.5 ± 5.1 ms (t_0_) to 55.9 ± 8.3 ms (t_3_) (*p* < 0.001, entire cartilage sample), from 42.9 ± 5.8 ms (t_0_) to 67.2 ± 19.7 ms (t_3_) (*p* < 0.001, superficial layer), and from 27.7 ± 6.0 ms (t_0_) to 44.4 ± 11.4 ms (t_3_) (*p* < 0.001, deep layer). Significant post hoc differences were found between t_0_ and t_1_ (except for deep layer), t_0_ and t_2_, and t_0_ and t_3_ (Supplementary Table S1).

At individual time points, group-wise, i.e., impaction energy-related, differences in T2 were significant primarily in the superficial layers at t_1_, t_2_, and t_3_ (Table 4). Post hoc analysis revealed the differences to be significant when comparing controls and HIMP-exposed samples, indicating significantly higher T2 values after HIMP exposure than in controls (Supplementary Table S2). Relative changes of T2 values confirmed these findings (Table 5). When considering the entire cartilage sample, the magnitude of relative changes gradually increased as a function of time, i.e., Δ_1_ < Δ_3_, and impaction energy level, i.e., CONT < LIMP < HIMP. Statistical analysis revealed these changes to be significant for Δ_1_ and Δ_3_ only, while Δ_2_ tended towards significance in all ROIs. Post hoc analysis indicated that the differences were significant only for the CONT vs. HIMP comparison, while CONT vs. LIMP or LIMP vs. HIMP were not significant (Supplementary Table S3).

### 3.4. MRI Data—Texture Feature Analysis

Radiomic texture features were significantly different only after HIMP exposure, and not in controls or after LIMP exposure (Table 6). Metrics of contrast displayed distinct changes. Contrast values were undulating in controls, while after LIMP exposure, samples’ contrast values tended to increase from 0.25 ± 0.17 (t_0_) to 0.28 ± 0.11 (t_3_) (*p* = 0.270). After HIMP exposure, however, contrast values were significantly increased from 0.24 ± 0.09 (t_0_) to 0.40 ± 0.17 (t_3_) (*p* < 0.001). Opposite observations were made for homogeneity that remained relatively constant in controls (*p* = 0.615) and tended to decrease in LIMP-exposed samples (*p* = 0.166) but decreased significantly from 0.88 ± 0.05 (t_0_) to 0.82 ± 0.06 (t_3_) (*p* < 0.001) in HIMP-exposed samples. Energy as a metric of orderliness was characterized by distinct changes, too. While energy was undulating in controls (*p* = 0.510), it decreased considerably from 0.43 ± 0.20 (t_0_) to 0.30 ± 0.07 (t_3_) (*p* = 0.016) and from 0.35 ± 0.13 (t_0_) to 0.24 ± 0.16 (t_3_) (*p* = 0.006) after LIMP and HIMP exposure, respectively, thereby tending towards (LIMP) or reaching statistical significance (HIMP). Variance as a statistical metric was undulating, too, in controls, while it underwent moderate, yet non-significant increases from 111.0 ± 94.9 (t_0_) to 143.2 ± 69.4 (t_3_) (*p* = 0.015) following LIMP exposure and strong and significant increases from 118.3 ± 79.9 (t_0_) to 311.4 ± 264.8 (t_3_) (*p* = 0.005) following HIMP exposure. Group-wise comparisons at individual time points revealed no significant differences at t_0_, indicating largely similar baseline texture feature and sample homogeneity prior to exposure. For contrast, homogeneity, and energy, significant group-wise differences were found at t_2_ and t_3_, while for variance, significant differences were only observed at t_3_.

### 3.5. MRI Data—Image Evaluation

**Figure 4:**
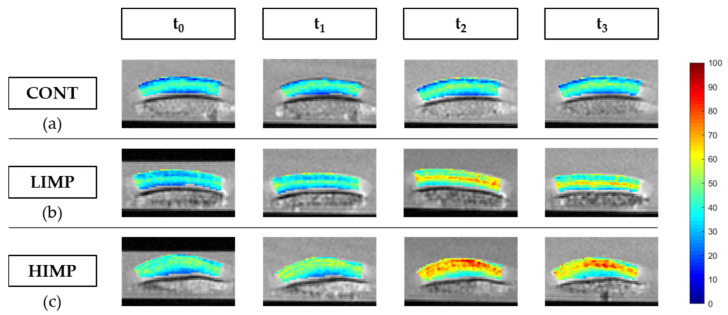
Color-coded and spatially resolved T2 parameter maps of representative cartilage samples and their post-impaction changes. Control samples without impaction (**a**). Cartilage samples displayed distinct changes after low-energy impaction (**b**) and more pronounced and widespread changes after high-energy impaction (**c**). Time points are marked by t_0_, t_1_, t_2_, and t_3_ that indicate the pre- (t_0_) and post-impaction measurements (t_1_) as well as those after 24 h (t_2_) and 72 h (t_3_). Scale bar on the right indicates color-coded T2 values [ms].

Impaction-induced quantitative changes as outlined above were reflected by corresponding qualitative changes in the T2 maps (Figure 4). Controls remained largely unchanged and maintained the inherent depth-wise stratification of T2 values with lower values in deeper and higher values in the more superficial cartilage zones. After LIMP exposure, the typical depth-wise stratification was progressively lost and gradually replaced by a band-like hyperintense signal zone at the sample centre. The hyperintense zone was not confined to the area of impaction, extended throughout the entire sample width, and was oriented parallel to the subchondral lamella. After HIMP exposure, the depth-wise stratification was lost immediately and superseded by a more diffuse and widespread area of hyperintense signal that gradually increased in size and hyperintensity to eventually involve the large parts of the sample’s cross-sectional area. Once settled, this area remained largely constant in the mid-to-long term. Supplementary Figure S1 gives more representative cartilage samples and their post-impaction changes.

## 4. Discussion

The most important finding of this study is that advanced MRI acquisition and postprocessing techniques, i.e., quantitative T2 mapping and texture feature analysis, may be used to (i) differentiate the severity of supraphysiological impact injuries of cartilage and (ii) monitor post-traumatic degenerative changes.

Prior to its initiation, this study had been motivated by the **lack of basic research** available on the association of T2 mapping and traumatic cartilage injury. Even though a solid body of clinical evidence is available to support the potential of T2 mapping in evaluating posttraumatic cartilage changes [17], the changes in T2 related to traumatic injury are variable and inconsistent. In young adults with recurrent patellar dislocations during childhood, significant decreases in T2 were found in the superficial patellar cartilage zone of injured as compared to non-injured joints, which may be an early sign of cartilage pathology [18]. After ACL reconstruction, inconsistent T2 changes relative to uninjured controls were found. At the one-year follow-up, T2 was not significantly elevated in one study [19], while others found significant T2 elevations at the medial femoral cartilage after two [20] and three years [21]. In contrast, increases in T2 were associated with morphologic cartilage lesions [22] as well as morphologically intact cartilage that is going to develop morphologic cartilage lesions in the years to come [23]. This, of course, indicates that compositional changes -as assessed by T2 mapping- precede the development of morphologic cartilage lesions and underscores the potential of T2 mapping to identify cartilage regions at risk of incipient degeneration.

This study clearly demonstrates that **injurious cartilage impaction** is associated with increasing T2 values as a function of impaction energy level and time. Based on the sensitivity profile of T2 versus structural and compositional cartilage properties, these increases reflect numerous single-impact-associated posttraumatic changes in cartilage [32,41,42,48,49,50,51]. The literature data indicate that these changes include surface damage, loss of proteoglycans and collagen network integrity, as well as chondrocyte death. These changes closely resemble degenerative changes in OA, are therefore often referred to as traumatic OA-like changes [42], and provide the degenerative correlates of altered T2 values. For the sake of comparability, impaction energy levels were chosen in line with earlier studies and, mechanistically, the induction of cartilage damage by dropping weights from defined heights has been thoroughly validated before [32,41,48]. Nonetheless, in this study, histologic reference indicated impaction-energy-associated surface disintegration and incipient-to-moderate proteoglycan depletion, thereby confirming the mode of action within the framework of this study.

On the tissue level, the impaction-induced **increases in T2** may be secondary to numerous posttraumatic changes that are excellently reviewed in [52]. For once, proteoglycans (and -in parts- collagen) are lost secondary to the collagen network damage. Lower proteoglycan and collagen contents are associated with higher T2 values [53]. For another, collagen network disintegration is induced directly by mechanical disruption and indirectly by subsequent enzymatic degradation. These processes contribute to increased tissue water content and tissue swelling, which are associated with higher T2 values, too [33], as well as increased collagen fibre disorientation and anisotropy. Secondary to impaction, the percentage of fibres oriented at magic angle, i.e., at 55° to the main magnetic field, may be elevated, thereby increasing T2 values, too [54]. Yet, even though these mechanisms are plausible, it remains unclear which exact compositional or (ultra)structural mechanism is primarily behind the prominent increases in T2.

Beyond mean T2 values, this study focused on **radiomic texture features**, too, as refined imaging biomarkers of cartilage trauma. Again, changes were clear and significant after HIMP exposure, while they were moderate and only tended towards significance after LIMP exposure. Across the spectrum of texture features assessed, variance and metrics of contrast and orderliness were significantly increased (i.e., contrast, variance) and decreased (i.e., homogeneity, energy) as a function of impaction energy. Additionally, these features were different between the various time points, indicating lower textural uniformity and growing structural disorder. These changes may be considered a sign of cartilage damage, too, as described before [25,26,44].

Notably, changes in T2, i.e., absolute values and texture features, were subject to gradual and drastic **changes over time**. These aspects may be explained by the concurrence of posttraumatic changes in cartilage that are induced either immediately through the impaction itself or delayed through cell death and the induction of matrix-degrading enzymes. These processes reduce biosynthetic capacity or bring about progressive degradation that may explain the gradual alterations in T2 characteristics. Most likely, these processes are at the root of the hyperintense bands that traversed the cartilage sample in the transitional zone parallel to the subchondral lamella. Observed after both low- and high-energy exposure, similar histologic changes have been reported before [32,41]. Following impaction of bovine cartilage, Jeffrey et al. found horizontal fissures in the transitional zone that they hypothesized to be due to deflection of the extracellular matrix with partial delamination of the upper and lower tissue portions. Other studies also demonstrated sub-surface intra-tissue damage and chondrocyte death prior to surface disintegration [55]. The band’s progression in terms of size and signal characteristics was clearly associated with impaction energy level and may thus reflect the ongoing structural and compositional changes of traumatized cartilage.

Notably, **zonal changes** of the superficial and deep tissue layers were roughly similar in terms of relative changes in T2. Considering the direct impaction of the cartilage surface, intuitively, one would expect larger changes of superficial than deeper zones. Yet, the viscoelastic nature of cartilage, its unique compressive properties and tight attachment to the underlying subchondral bone provide efficient mechanisms for absorption of physiologic and supraphysiologic loads throughout the entire tissue depth [56]. Once the subchondral bone is removed, these mechanisms of load distribution and dissipation are disrupted and the protective effect is lost [41].

This study has numerous **limitations**. First, the experimental in vitro design necessitated excision and preparation of cartilage and its prolonged incubation, thereby limiting the clinical translatability of our findings. Prolonged incubation in media may artificially increase tissue hydration, thereby increasing T2 values. As this was observed for otherwise unaffected control samples, these gradual increases in T2 provide the background against which the impaction-induced changes must be considered. Additionally, scanning was performed at room temperature, again affecting T2 values. As the MRI measurements were performed in a standardized manner, this bias may be considered systematic. Additional studies using in situ human whole-knee joint configurations and more physiologic impaction methods are thus required to confirm our findings. As an additional caveat, however, resultant intra-tissue changes secondary to impaction are largely dependent on the experimental framework conditions. Standardized impaction of cartilage samples induces more severe changes than similar impaction of intact joints [57]. Second, human cartilage samples were obtained from knee joints undergoing joint replacement. Despite our best efforts to ascertain tissue quality by macroscopic evaluation and baseline histology, the pre-existent degeneration of the tissue brought about by chronic mechanical and inflammatory disease processes is clearly uncontrolled and may have altered the tissue’s susceptibility to impaction loading. Even though the longitudinal study design allows for consistent intra-sample referencing and reduces this type of bias, tissue variability clearly affects tissue susceptibility and outcomes of impaction. Future studies should therefore use ‘truly’ healthy cartilage through alternative tissue sources such as amputations or organ-donor networks to realize improved tissue quality control. Third, despite selecting cartilage from the lateral femoral condyles for reasons of topoanatomic consistency, human cartilage thickness is largely different between individuals [58] and may affect load distribution and dissipation in the tissue. Fourth, the cartilage samples surfaces were oriented parallel to the main magnetic field B0 and, consequently, the majority of collagen fibres of the deep and transitional zones were oriented at 90° to B0, thereby affecting T2 quantification. T2 values may increase due to the magic angle effect as collagen fibre orientation changes relative to B_0_ after injurious impaction, particularly in the deep tissue layer [54] Although measurement conditions were standardized (using a dedicated device) and inter-sample variability in position and configuration thus decreased, this aspect needs to be considered, too, prior to any clinical translation. Fifth, this study only focused on T2 mapping, while other imaging markers, e.g., T1ρ, T1, T2 *, sodium, gagCEST (glycosaminoglycan Chemical Exchange Saturation Transfer), or contrast-enhanced techniques such as dGEMRIC (delayed gadolinium-enhanced MRI of cartilage) are of potential value in assessing cartilage structure and composition [14,34,38,59,60,61,62,63,64]. Of these, T1ρ mapping is of additional and complementary value to T2 mapping because of distinct biophysical properties [65]. Yet, the exact sensitivity profile of T1ρ remains to be determined with proteoglycan, collagen, and water content as well as collagen fibre orientation potentially contributing to T1ρ relaxation characteristics. As of today, T1ρ seems to indicate the cartilage tissue’ macromolecular configuration [53,66,67,68,69]. In posttraumatic contexts, the sensitivity of T1ρ to changes in the tissue’s solid and fluid constituents and its mechanical condition [38,70,71,72,73] may be of value in future pre-clinical and clinical studies. Sixth, this study did not include dedicated compositional reference measures and, consequently, no advanced quantification of proteoglycan or collagen content that would have allowed spatially resolved associations of T2 maps and compositional measures. Prior to any clinical translation, these associations ought to be clarified in posttraumatic contexts and beyond.

## 5. Conclusions

This study demonstrates that advanced MRI acquisition and postprocessing techniques, i.e., quantitative T2 mapping and texture feature analysis, are sensitive diagnostic means to detect and monitor traumatic impaction injuries of cartilage and associated posttraumatic degenerative changes. If corroborated by additional in situ and in vivo studies, the close association of changes in T2 and texture features, impaction energy level, and time render this technique diagnostically promising to assess cartilage tissue after trauma and to detect cartilage at risk.

## Figures and Tables

**Table 1:** Details as a function of impaction characteristics.

Group	Mass (g)	Height (mm)	Velocity (m/s)	Energy (J)
Low Impact (LIMP)	500	100	1.4	0.49
High Impact (HIMP)	1000	100	1.4	0.98

**Table 2:** Acquisition parameters of MR sequences.

Parameters	T2 Map	PD-Weighted
Sequence type	multi-spin echo	turbo-spin echo
Orientation	mid-sag ^1^	ax, cor, sag ^2^
Repetition time [ms]	1500	1500–1589
Echo time [ms]	*n* × 8.38 (*n* = 1–12)	11
Turbo spin echo factor [n]	12	6
Field of view [mm]	52 × 52	62 × 62
Acquisition matrix [pixels]	176 × 176	144 × 142
Reconstruction matrix [pixels]	224 × 224	256 × 256
Pixel size [mm/pixel]	0.23 × 0.23	0.24 × 0.24
Flip angle [°]	90	90
Number of signal averages [n]	2	2
Slices [n]	1	8–24
Slice thickness [mm]	2.0	1.0
Slice gap [mm]	-	0.5
Duration [min sec]	4 min 29 s	7 min 11 s ^3^

^1^ mid-sag—mid-sagittal, ^2^ ax—axial, cor—coronal, sag—sagittal, ^3^ total duration of all three sequences.

**Table 3:** Histologic changes in cartilage samples 72 h after impaction (LIMP, HIMP) or incubation under control conditions. Based on the Mankin classification [47], surface integrity (score 0–6) and proteoglycan staining intensity (score 0–4) scores are indicated as absolute changes relative to baseline. Group-wise differences were assessed using one-way ANOVA tests. CONT—controls, LIMP—low impaction energy, HIMP—high impaction energy.

Histologic Cartilage Change	CONT	LIMP	HIMP	*p*-Value
Surface Integrity	0.14 ± 0.34	0.43 ± 0.82	1.14 ± 1.4	0.100
Proteoglycan Staining Intensity	0.14 ± 1.25	0.64 ± 1.17	0.93 ± 1.03	0.163

**Table 4:** Mean absolute T2 values as a function of region-of-interest, time point, and impaction energy level. T2 values are given as means ± standard deviation [ms] for the entire cartilage sample as well as the respective superficial and deep tissue layers. Longitudinal (time-related) differences in T2 values were assessed by the Friedman test and respective p-values are organized in columns and indicated by (§). Cross-sectional (group-related) differences in T2 values at individual time points were assessed using the Kruskal–Wallis test and respective p-values are organized in lines and indicated by (‡). Post hoc test details are given in Supplementary Table S1. CONT—controls, LIMP—low-energy impaction, HIMP—high-energy impaction. Significant differences are indicated in **bold type**.

Region-of-Interest	Group	Time				*p*-Value (§)
		t_0_	t_1_	t_2_	t_3_	
**Entire Cartilage Sample**	**CONT**	33.3 ± 5.1	34.4 ± 6.1	35.5 ± 6.2	35.1 ± 7.4	0.093
	**LIMP**	32.0 ± 2.4	33.8 ± 3.6	38.7 ± 4.5	40.3 ± 5.2	**<0.001**
	**HIMP**	35.5 ± 5.1	40.8 ± 5.9	45.4 ± 8.3	55.9 ± 8.3	**<0.001**
	***p*-value (‡)**	0.234	0.016	0.019	**0.002**	
**Superficial Layer**	**CONT**	37.0 ± 6.4	39.1 ± 7.6	39.5 ± 7.2	39.0 ± 8.8	0.180
	**LIMP**	38.2 ± 4.3	42.4 ± 5.2	46.3 ± 4.4	47.5 ± 6.1	**<0.001**
	**HIMP**	42.9 ± 5.8	48.8 ± 6.3	54.5 ± 9.7	67.2 ± 19.7	**<0.001**
	***p*-value (‡)**	0.026	**0.008**	**0.001**	**0.001**	
**Deep Layer**	**CONT**	29.4 ± 5.1	29.4 ± 5.1	31.4 ± 6.2	31.1 ± 6.6	0.18
	**LIMP**	26.1 ± 2.8	28.1 ± 3.8	32.8 ± 7.7	33.9 ± 6.9	**<0.001**
	**HIMP**	27.7 ± 6.0	32.6 ± 7.1	36.2 ± 9.2	44.4 ± 11.4	**<0.001**
	***p*-value (‡)**	0.247	**0.008**	0.557	0.011	

**Table 5:** Relative changes of T2 values as a function of region-of-interest, time point, and impaction energy level. Relative changes (Δ) were calculated as Δ_x_ = ((T2t_x_/T2t_0_) − 1) × 100 [%] for the time points t_1_, t_2_, and t_3_. For each time point, Δ values were compared in a group-wise manner based on one-way ANOVA tests. Significant differences are indicated in **bold type**. For an explanation of the abbreviations please refer to Table 4.

Region of Interest	Group	Δ_1_	Δ_2_	Δ_3_
**Entire Cartilage Sample**	**CONT**	3.0 ± 3.4	6.7 ± 5.4	4.7 ± 6.5
	**LIMP**	9.5 ± 3.5	23.7 ± 17.6	27.1 ± 16.4
	**HIMP**	15.3 ± 8.3	28.6 ± 16.1	59.2 ± 42.1
	***p*-value**	**<0.001**	0.012	**<0.001**
**Superficial Layer**	**CONT**	5.5 ± 5.1	6.8 ± 6.3	4.9 ± 8.8
	**LIMP**	10.9 ± 11.0	22.5 ± 23.9	25.1 ± 27.2
	**HIMP**	14.0 ± 14.2	27.6 ± 29.3	57.1 ± 61.5
	***p*-value**	**0.002**	0.041	**0.003**
**Deep Layer**	**CONT**	0.4 ± 5.9	6.7 ± 9.0	−1.2 ± 5.1
	**LIMP**	7.8 ± 7.7	25.4 ± 27.0	29.9 ± 31.4
	**HIMP**	18.4 ± 18.0	30.9 ± 32.3	63.6 ± 67.1
	***p*-value**	0.013	0.019	**<0.001**

**Table 6:** Absolute values of radiomic texture features as a function of impaction energy level and time point. Values are given as means ± standard deviation for the entire cartilage samples. Please see Table 4 for details on table organization, statistical analysis, and abbreviations. P-values of time-related differences in T2 values (Friedman test) are organized in columns (§) and p-values of group-related differences in T2 values (Kruskal–Wallis test) are organized in lines (‡). Significant differences are indicated in **bold type**.

Texture Feature Class	Texture Feature	Groups	Time				*p*-Value (§)
			t_0_	t_1_	t_2_	t_3_	
**Metrics of Contrast**	**Contrast**	**CONT**	0.18 ± 0.07	0.19 ± 0.06	0.18 ± 0.04	0.17 ± 0.06	0.615
		**LIMP**	0.25 ± 0.17	0.27 ± 0.14	0.26 ± 0.08	0.28 ± 0.11	0.270
		**HIMP**	0.24 ± 0.09	0.30 ± 0.09	0.30 ± 0.09	0.40 ± 0.17	**<0.001**
		***p*-value (‡)**	0.312	0.06	**0.003**	**0.001**	
	**Homogeneity**	**CONT**	0.91 ± 0.03	0.91 ± 0.02	0.91 ± 0.02	0.91 ± 0.03	0.615
		**LIMP**	0.89 ± 0.05	0.87 ± 0.05	0.87 ± 0.03	0.87 ± 0.04	0.166
		**HIMP**	0.88 ± 0.05	0.86 ± 0.04	0.86 ± 0.03	0.82 ± 0.06	**0.001**
		***p*>-value (‡)**	0.256	0.074	**0.003**	**0.001**	
**Metric of Orderliness**	**Energy**	**CONT**	0.49 ± 0.19	0.46 ± 0.17	0.46 ± 0.11	0.48 ± 0.15	0.510
		**LIMP**	0.43 ± 0.20	0.36 ± 0.16	0.32 ± 0.07	0.30 ± 0.07	0.016
		**HIMP**	0.35 ± 0.13	0.30 ± 0.10	0.28 ± 0.10	0.24 ± 0.16	**0.006**
		***p*-value (‡)**	0.225	0.078	**0.004**	**0.002**	
**Statistical Metric**	**Variance**	**CONT**	69.5 ± 28.7	77.4 ± 33.0	70.5 ± 27.1	67.1 ± 29.4	0.392
		**LIMP**	111.0 ± 94.9	142.1 ± 92.6	128.2 ± 58.6	143.2 ± 69.4	0.015
		**HIMP**	118.3 ± 79.9	157.3 ± 101.6	189.7 ± 124.6	311.4 ± 264.8	**0.005**
		***p*-value (‡)**	0.277	0.118	0.014	**0.010**	

## Data Availability

The raw data presented in this study are available from the corresponding author upon reasonable request.

## Supplementary Materials

The following are available online at https://www.mdpi.com/2075-1729/11/3/201/s1, Figure S1: Additional examples of color-coded and spatially resolved T2 parameter maps of representative cartilage samples and their post-impaction changes. Table S1: Post hoc details of absolute T2 values as a function of time-point-wise comparisons, Table S2: Post hoc details of absolute T2 values as a function of group-wise comparisons, Table S3: Post hoc comparisons of relative changes.
