# Supplementary material for: Longitudinal T2 Mapping and Texture Feature Analysis in the Detection and Monitoring of Experimental Post-Traumatic Cartilage Degeneration
Source: Life (Basel). 2021 Mar 5;11(3):201. doi: 10.3390/life11030201 (PMC8000874; doi:10.3390/life11030201)
Supplement: Supplementary file 1 [file life-11-00201-s001.pdf]

Supplementary Material

Title: Longitudinal T2 Mapping and Texture Feature Analysis in the Detection and Monitoring of Experimental Post-Traumatic Cartilage Degeneration

Authors: Marc Sebastian Huppertz, Justus Schock, Karl Ludger Radke, Daniel Benjamin Abrar, Manuel Post, Christiane Kuhl, Daniel Truhn and Sven Nebelung

Journal: Life

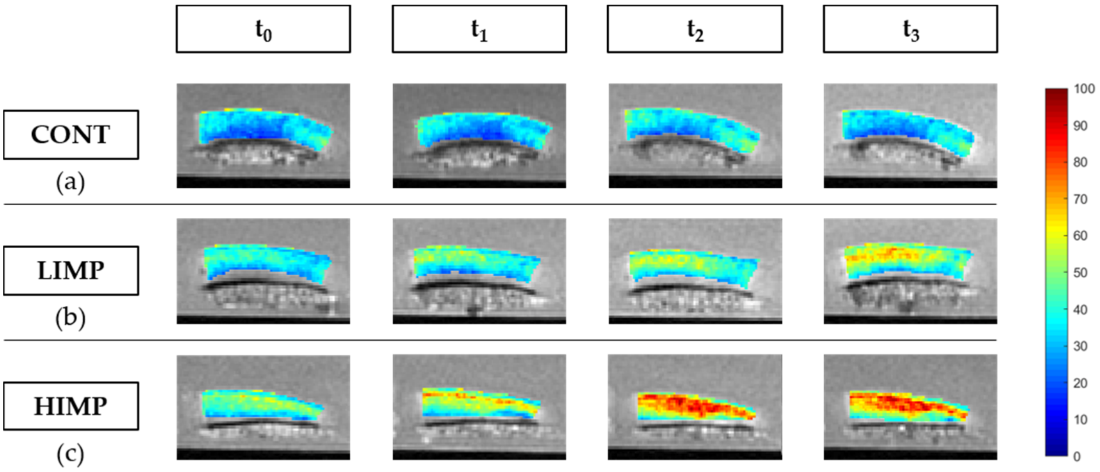

Supplementary Figure S1. Additional examples of color-coded and spatially resolved T2 parameter maps of representative cartilage samples and their post-impaction changes. See **Figure 4** for details on groups and time lines as well as abbreviations.

Supplementary Table S1. Dunn's multiple comparison post-hoc details of time-related pair-wise comparisons of absolute T2 values, i.e., t_0 to t_3, and as a function of impaction energy level. Respective p-values indicate post-hoc results of pair-wise comparisons in the entire cartilage sample (entire) and the superficial and deep tissue layers. LIMP – low-energy impaction, HIMP – high-energy impaction. Significant differences are printed in **bold type**.

		**Region of Interest**		
**Group**	**Comparison**	**entire**	**superficial**	**deep**
**LIMP**	**t_0 vs. t_1**	0.021	0.033	0.051
	**t_0 vs. t_2**	**<0.001**	**0.002**	**<0.001**
	**t_0 vs. t_3**	**<0.001**	**<0.001**	**<0.001**
	**t_1 vs. t_2**	1.000	1.000	0.859
	**t_1 vs. t_3**	0.474	1.000	0.115
	**t_2 vs. t_3**	1.000	1.000	1.000
**HIMP**	**t_0 vs. t_1**	**0.008**	**0.005**	0.013
	**t_0 vs. t_2**	**<0.001**	**<0.001**	**<0.001**
	**t_0 vs. t_3**	**<0.001**	**<0.001**	**<0.001**
	**t_1 vs. t_2**	1.000	1.000	1.000
	**t_1 vs. t_3**	0.859	1.000	0.474
	**t_2 vs. t_3**	1.000	1.000	1.000

Supplementary Table S2. Dunn's multiple comparison post-hoc details of group-related pair-wise comparisons of absolute T2 values at distinct time points as a function of region-of-interest. Given are the p-values for pair-wise group comparisons at distinct time points, i.e., t0 – t3. Significant differences are printed in **bold type**. Please refer to **Supplementary Table S1** (above) for an explanation of the abbreviations.

Region-of-Interest	Comparison	t_0	t_1	t_2	t_3
**Entire cartilage sample**	**CONT vs. LIMP**	1.000	1.000	0.739	0.526
	**CONT vs. HIMP**	1.000	0.075	0.022	**0.003**
	**LIMP vs. HIMP**	0.269	0.033	0.1875	0.050
**Superficial layer**	**CONT vs. LIMP**	1.000	1.000	0.250	0.312
	**CONT vs. HIMP**	0.069	0.017	**0.001**	**0.001**
	**LIMP vs. HIMP**	0.073	0.043	0.081	0.067
**Deep layer**	**CONT vs. LIMP**	0.284	1.000	1.000	1.000
	**CONT vs. HIMP**	0.795	1.000	1.000	0.034
	**LIMP vs. HIMP**	1.000	0.180	1.000	0.036

Supplementary Table S3. Tukey's multiple comparison post-hoc details of pair-wise comparisons of relative changes in T2 values Δ_x as a function of region-of-interest and pair-wise group comparison. Δ_x refers to the relative change at time point t_x and is calculated by $\Delta_x = ((T2t_x/T2t_0) - 1) * 100$ [%]. Significant differences are printed in **bold type**. Please refer to **Supplementary Table S1** (above) for an explanation of the abbreviations.

		Region-of-Interest		
**Relative Change**	**Comparison**	**entire**	**superfi-cial**	**deep**
	**CONT vs. LIMP**	0.055	0.048	0.447
**Δ_1**	**CONT vs. HIMP**	**<0.001**	**0.001**	0.014
	**LIMP vs. HIMP**	0.045	0.204	0.092
	**CONT vs. LIMP**	0.052	0.132	0.070
**Δ_2**	**CONT vs. HIMP**	**0.0097**	0.033	0.015
	**LIMP vs. HIMP**	0.691	0.704	0.687
	**CONT vs. LIMP**	0.215	0.374	0.085
**Δ_3**	**CONT vs. HIMP**	**<0.001**	**0.004**	**<0.001**
	**LIMP vs. HIMP**	0.019	0.033	0.017
